# Primary cutaneous adenoid cystic carcinoma of the right forearm: a case report and dermoscopic features

**DOI:** 10.3389/fmed.2026.1872296

**Published:** 2026-06-16

**Authors:** Si Li, Xue Cheng, Ronggui Xing, Zhenyin Peng, Tianyou Xiong, Yanan Jiang, Xianbiao Zou

**Affiliations:** Department of Dermatology, South China Hospital, Medical School, Shenzhen University, Shenzhen, China

**Keywords:** case report, dermatologic surgery, dermoscopy, pathology, primary cutaneous adenoid cystic carcinoma

## Abstract

Primary cutaneous adenoid cystic carcinoma (PCACC) is a rare, slow-growing malignant adnexal tumor of the skin first described in the 1970s. We present the case of a 63-year-old man who developed a slow-growing nodule on the right forearm, an atypical location for this tumor. The diagnosis of PCACC was confirmed by histopathological examination. This report discusses the pathological, dermoscopic, and radiological characteristics of the lesion, as well as its successful management with wide local excision. This case underscores the importance of considering PCACC in the differential diagnosis of slow-growing cutaneous nodules, even when it occurs at unusual sites. While dermoscopy can provide valuable diagnostic clues, a definitive diagnosis necessitates histopathological evaluation. Wide local excision remains the standard therapeutic approach to minimize the risk of local recurrence, and long-term follow-up is imperative due to the potential for delayed recurrence.

## Introduction

Primary cutaneous adenoid cystic carcinoma (PCACC) is a rare cutaneous malignancy first described in the 1970s. It predominantly occurs in middle-aged and elderly individuals, with no significant gender difference in incidence (1, 2). The most common sites are the head, face, and neck, but it can also arise in other body regions (3). Clinically, it presents as a slowly enlarging, painless, fixed subcutaneous nodule or plaque. The tumor exhibits neurotropism, often spreading along nerve fibers, and is characterized by a high rate of local recurrence but low lymph node involvement.

The diagnosis of PCACC requires exclusion of direct extension from salivary gland adenoid cystic carcinoma and rare metastatic tumors to the skin (4). Typical histopathological features include the following: the tumor has no connection with the epidermis; it is located in the dermis and displays a diffuse infiltrative growth pattern; perineural invasion is present; and the tumor nests are composed of adenoid or cribriform patterns together with many small solid epithelial cell clusters. Immunohistochemical findings show positive expressions of CK, CEA, EMA, and CD117 (5).

## Case presentation

We present the case of a 63-year-old man who presented with a red plaque on his right forearm that had been present for over 10 years. Initially, the lesion appeared as a black macule the size of a soybean, occasionally scratched by the patient, but he never sought medical attention. Over the past 5 years, the lesion gradually enlarged and developed into a round to oval, yellow–red infiltrative plaque approximately 4 cm in diameter, with a hard consistency and mild tenderness to pressure. The patient reported a weight loss of 4 kg over the past 6 months but denied having a low-grade fever, night sweats, or other systemic symptoms. His past medical history was unremarkable, with no specific diseases. He denied any medication use or surgical history, as well as smoking and alcohol consumption. He had no history of occupational exposure, no prior trauma or radiation to the affected site, and no family history of malignancy.

**Figure 1:**
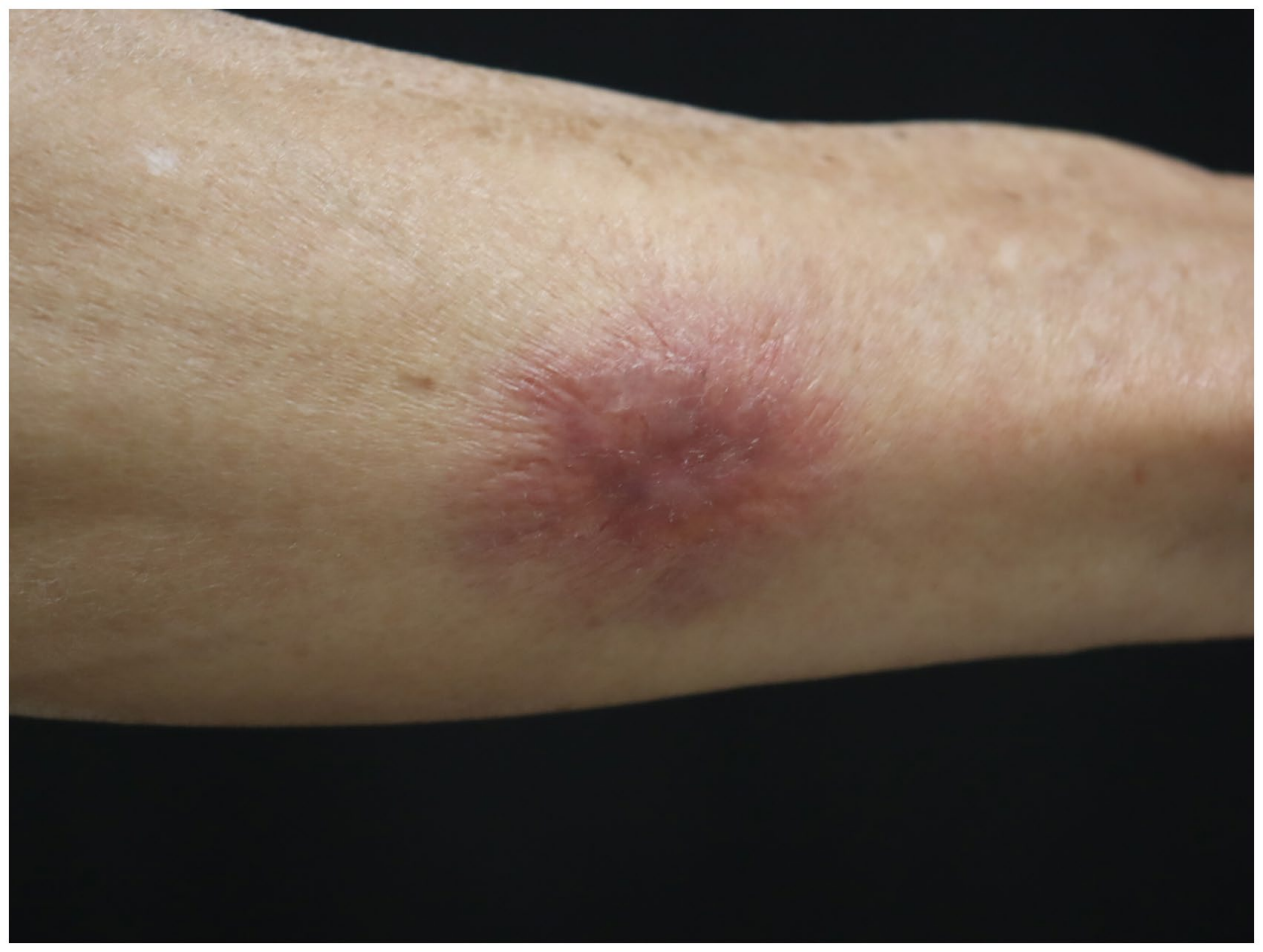
4-cm round yellow–red infiltrative plaque, smooth with central atrophy, ill-defined, hard, poor mobility, mildly tender.

Dermatological examination showed a round, yellow–red infiltrative plaque approximately 4 cm in diameter on the right forearm. The surface was smooth and slightly elevated above the skin level, with a central atrophic depression. The borders were ill-defined, the consistency was hard, mobility was poor, and mild tenderness was present on palpation (Figure 1).

## Diagnostic assessment

Laboratory findings showed that complete blood count, coagulation function, liver and kidney function, blood glucose, and blood lipids were all within normal limits. Electrocardiogram showed no abnormalities.

**Figure 2:**
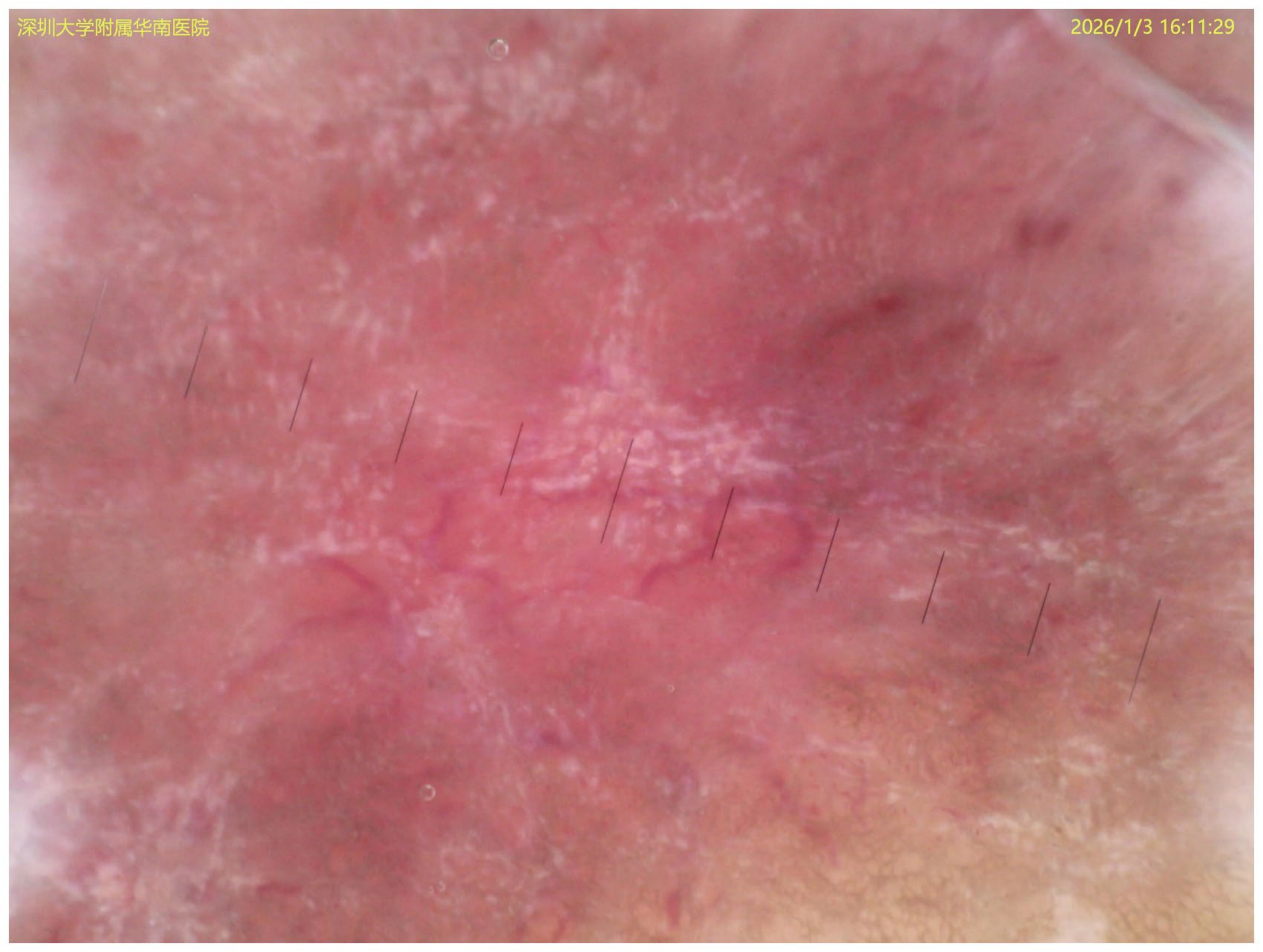
Dermoscopy: pink background, uniform linear vessels, short rod-like shiny white streaks, partially clustered and confluent, without arborizing vessels or no blue-gray ovoid nests.

Dermoscopy showed a pink background, uniform linear vessels, short rod-like shiny white streaks that were partially clustered and confluent, no arborizing vessels, and no blue–gray ovoid nests (Figure 2). Given these features, basal cell carcinoma (BCC) could not be entirely ruled out, prompting the recommendation for a skin biopsy and histopathological examination.

Color Doppler ultrasound of superficial masses revealed a hypoechoic area within the dermis and subcutaneous soft tissue of the right forearm, characterized by ill-defined borders and heterogeneous internal echogenicity. No significant blood flow signals were detected.

**Figure 3:**
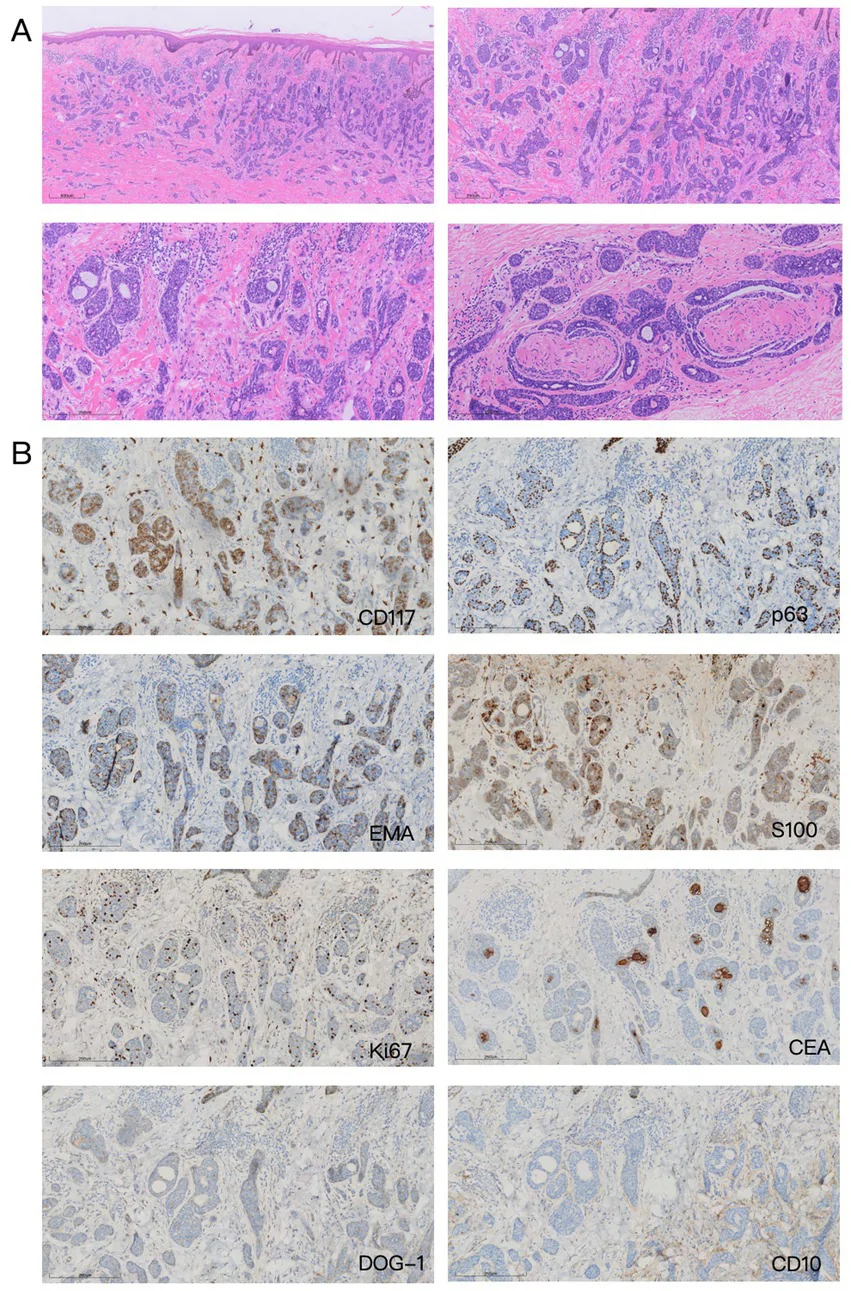
**(A)** Basophilic epithelial component forming ductal structures and cribriform patterns with perineural invasion (H&E, ×4, ×10, ×20). **(B)** CEA (focal, +), CD117 (+), EMA (partial, +), Ki67 (hot spot, 30%), p63 (basal layer, +), and s100(−) (×20).

Histopathology revealed that numerous tumor cells were observed within the dermis, growing in nests, comma-shaped structures, tubular structures, or cords. Eosinophilic homogeneous material was present within some tubular lumina. Tumor cell nests infiltrated into the deep dermis, and perineural invasion was observed (Figure 3A).

Immunohistochemistry showed CEA (focal+), CD117 (+), CD10 (−), EMA (partial+), Ki67 (hotspot 30%), DOG-1 (−), p63 (basal layer+), and S100 (−) (Figure 3B).

To rule out metastatic salivary gland ACC or advanced disease, we further performed imaging examinations. The results showed no abnormalities on high-resolution computed tomography (CT) of the head, neck, and chest, nor on whole-body bone scintigraphy.

The patient was diagnosed with primary cutaneous adenoid cystic carcinoma (PCACC).

### Therapeutic intervention and follow-up

**Figure 4:**
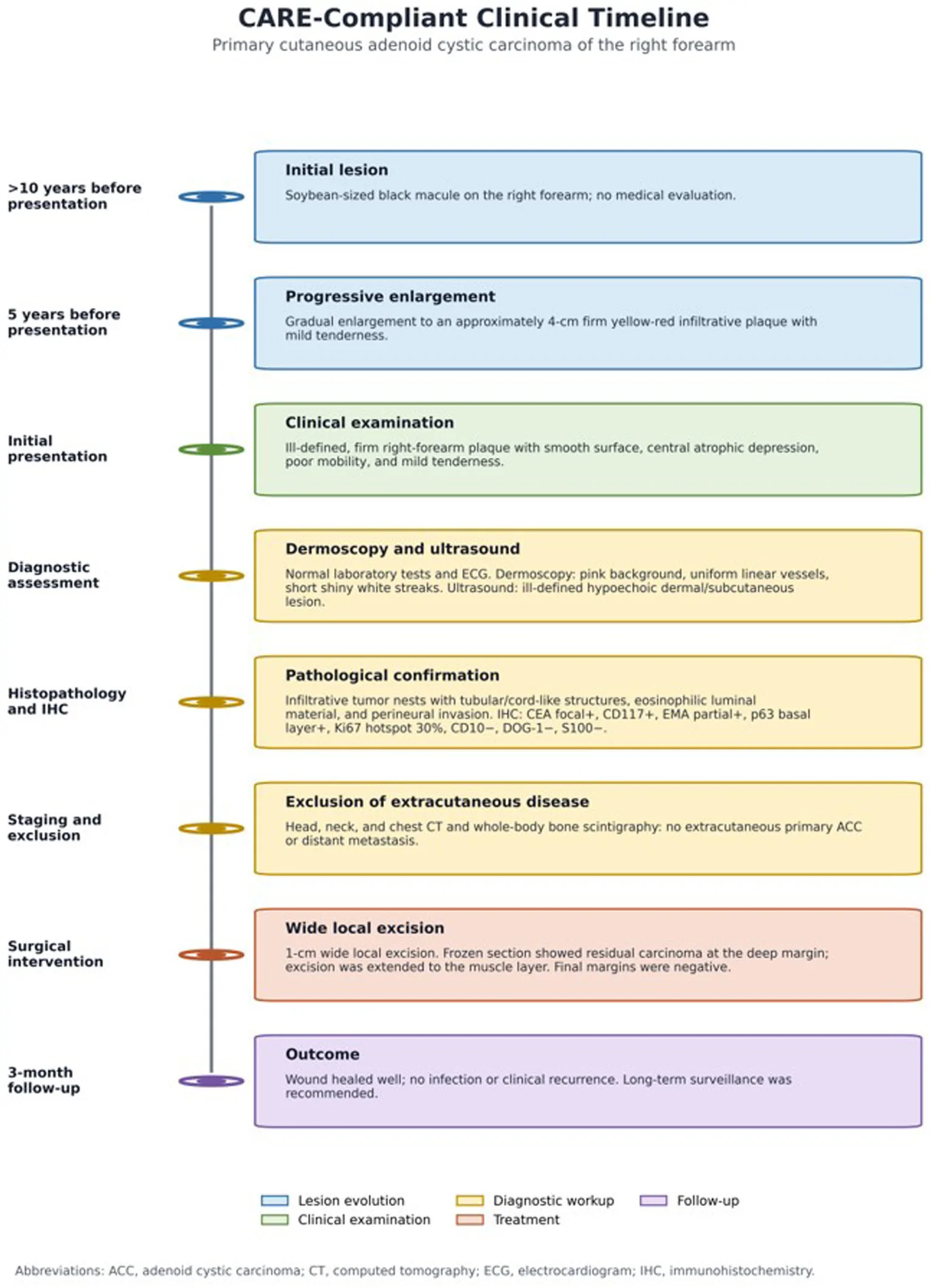
Clinical timeline summarizing lesion evolution, diagnostic workup, surgical intervention, and short-term follow-up.

Following routine preoperative evaluation, a 1-cm-wide local excision was performed along the premarked tumor margin. The specimen measured approximately 50 mm × 45 mm. Intraoperative frozen-section analysis showed residual carcinoma at the deep margin; therefore, excision was extended to the muscle layer. Meticulous hemostasis was achieved, followed by pressure dressing application. Primary closure was not feasible; thus, the wound was managed with open dressing changes. Final histopathology confirmed negative margins. At the 3-month follow-up, the wound had healed well with no signs of infection or tumor recurrence (Figure 4).

## Discussion

Primary cutaneous adenoid cystic carcinoma (PCACC) is an uncommon malignant adnexal tumor characterized by slow growth, infiltrative behavior, and a propensity for local recurrence (5, 6). Clinically, PCACC usually presents as a firm, indolent nodule or plaque that may be asymptomatic or associated with pain (7). Since these clinical features are non-specific, definitive diagnosis relies on histopathological examination and immunohistochemical correlation (8).

Histologically, PCACC typically shows cribriform and tubular architectural patterns composed of relatively monomorphic basaloid cells (5, 6). Mucin-containing pseudocystic spaces and true duct-like structures are important diagnostic clues (5, 8). Perineural invasion is a characteristic feature and has been associated with a higher risk of local recurrence and poorer clinical outcomes (7, 9). In this case, the tumor was located in the dermis and subcutaneous soft tissue, with perineural infiltration, supporting the malignant adnexal nature of the lesion. The diagnosis of PCACC requires careful exclusion of metastatic or directly extending adenoid cystic carcinoma, particularly from the salivary glands. This distinction is essential because primary cutaneous and extracutaneous adenoid cystic carcinomas may be morphologically similar and may share overlapping immunohistochemical profiles (7, 9). Therefore, diagnosis should not rely solely on routine histology or a single immunohistochemical marker. Instead, an integrated assessment of clinical history, imaging evaluation, tumor distribution, morphology, and immunophenotype is required. In this case, postoperative contrast-enhanced computed tomography of the head, neck, and chest did not reveal evidence of a salivary gland or visceral primary tumor, supporting the diagnosis of PCACC.

Immunohistochemically, PCACC usually demonstrates a biphasic ductal–myoepithelial phenotype. Luminal ductal cells may express CK7, EMA, CEA, and CD117/c-KIT, whereas abluminal myoepithelial cells may express p63, SMA, calponin, SOX10, or S100 with variable intensity (5, 6, 8). CD117 positivity supports adenoid cystic differentiation but is not specific and should be interpreted within the morphological context. In this case, CD117 positivity, EMA-supported ductal differentiation, cribriform/tubular architecture, and mucinous pseudolumina collectively supported the diagnosis (8). Immunohistochemical staining for DOG-1 and S100 was performed to support adenoid cystic differentiation and to aid in the differential diagnosis from adenoid cystic basal cell carcinoma, microcystic adnexal carcinoma, and other cutaneous adnexal tumors with cribriform or infiltrative growth patterns (8, 10, 11).

The main histopathological differential diagnoses include adenoid basal cell carcinoma, primary cutaneous cribriform carcinoma, cylindroma, and spiradenoma with adenoid cystic-like areas (7). Adenoid basal cell carcinoma is characterized by epidermal or follicular attachment, peripheral palisading, stromal retraction, mucinous stroma, and diffuse BerEP4 expression, whereas PCACC more often shows true bilayered ducts, mucin-filled pseudocysts, infiltrative growth, and myoepithelial differentiation. Primary cutaneous cribriform carcinoma may resemble PCACC because of its cribriform architecture, but it usually lacks a continuous ductal–myoepithelial cell population and perineural invasion. Cylindroma and spiradenoma usually retain their conventional architectural features, such as a jigsaw-puzzle arrangement or prominent intratumoral lymphocytes, and typically lack the diffuse malignant adenoid cystic pattern seen in PCACC (7, 8).

Dermoscopic descriptions of PCACC remain scarce. Reported findings include homogeneous erythematous nodules with arborizing vessels, erosions, and focal hemorrhagic areas, which may mimic basal cell carcinoma (12). In contrast, this case showed a pink background with uniform linear vessels and short rod-like shiny white streaks, without arborizing vessels or blue–gray ovoid nests. These observations suggest that PCACC may have heterogeneous and overlapping dermoscopic features. Dermoscopy is therefore best regarded as an adjunctive tool that may raise suspicion for cutaneous malignancy and support the decision to perform a biopsy or complete excision, rather than as a diagnostic modality for PCACC.

There is no standardized treatment protocol for PCACC because of its rarity. Complete surgical excision with histologically negative margins remains the mainstay of treatment. Regional lymph node dissection is generally reserved for patients with clinical or radiological evidence of nodal involvement (7, 9). Adjuvant radiotherapy may be considered in selected cases, such as positive margins, unresectable disease, extensive perineural invasion, or recurrence, although its role remains insufficiently defined (6, 7, 9, 13). In this case, wide local excision was performed as the primary treatment, and intraoperative frozen-section assessment allowed timely extension of the resection to achieve negative surgical margins. No evidence of tumor recurrence was observed during follow-up, supporting the effectiveness of complete margin-controlled excision for short-term disease control.

The major limitation of this report is the short follow-up period. Although no recurrence or metastasis was observed at 3 months after surgery, this interval is inadequate for evaluating the long-term behavior of PCACC. Late local recurrence and rare distant metastasis have been reported, even many years after initial treatment (6). Accordingly, long-term surveillance is essential, with careful assessment of the surgical scar, regional lymph nodes, and any symptoms suggestive of local recurrence or distant disease.

## Data Availability

The original contributions presented in the study are included in the article/Supplementary material, further inquiries can be directed to the corresponding author.
