# Correction: Primary cutaneous adenoid cystic carcinoma of the right forearm: a case report and dermoscopic features

**DOI:** 10.3389/fmed.2026.1926300

**Published:** 2026-08-31

**Authors:** Si Li, Xue Cheng, Ronggui Xing, Zhenyin Peng, Tianyou Xiong, Yanan Jiang, Xianbiao Zou

**Affiliations:** Department of Dermatology, South China Hospital, Medical School, Shenzhen University, Shenzhen, China

**Keywords:** case report, dermatologic surgery, dermoscopy, pathology, primary cutaneous adenoid cystic carcinoma

The images for Figures 1,2 were inadvertently swapped in their placement. The image currently displayed as Figure 1 should appear as Figure 2, and the image currently displayed as Figure 2 should appear as Figure 1. The correct image for the figures, and their respective captions, appears below.

**Figure 1:**
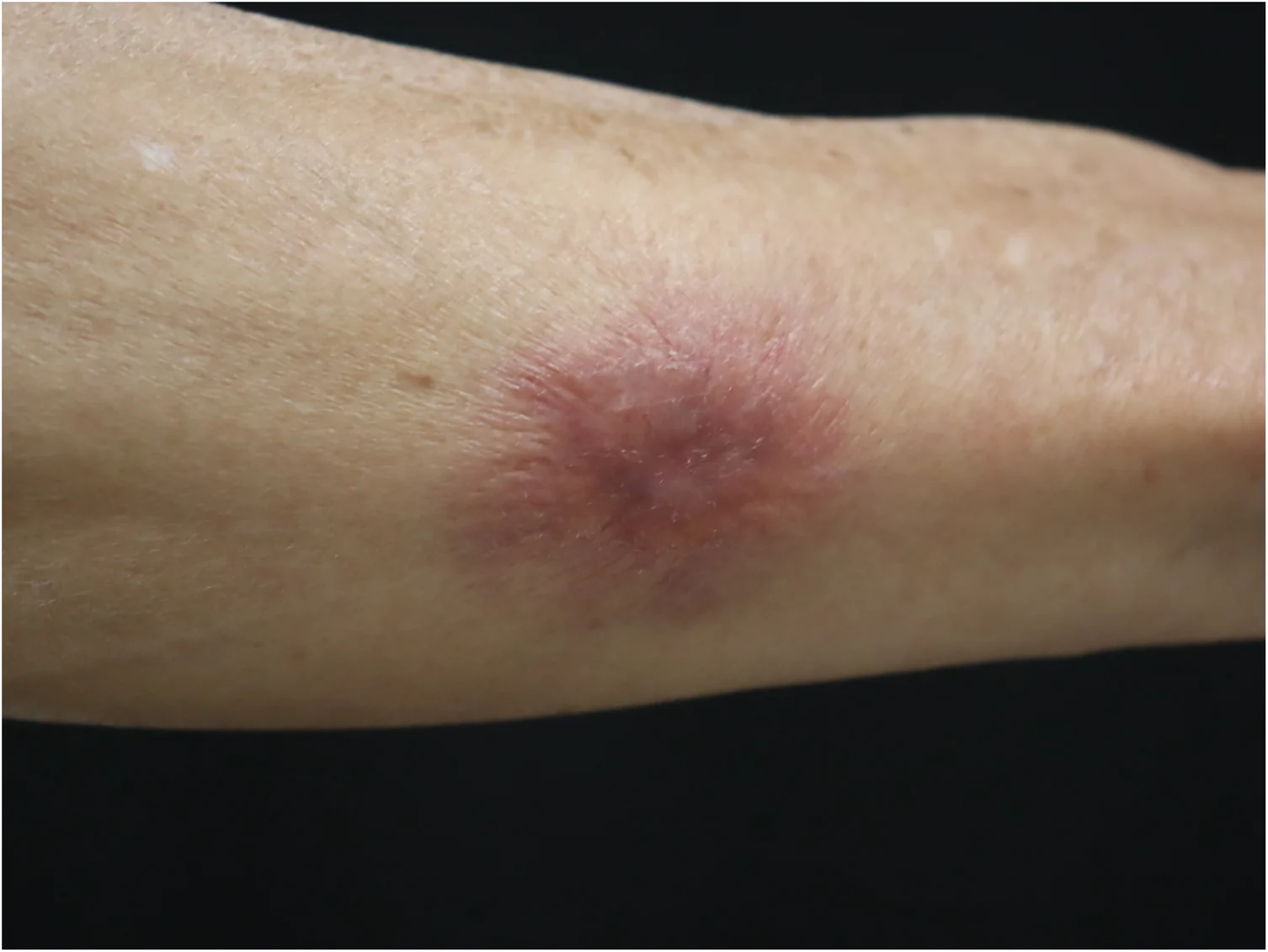
4-cm round yellow–red infiltrative plaque, smooth with central atrophy, ill-defined, hard, poor mobility, mildly tender.

**Figure 2:**
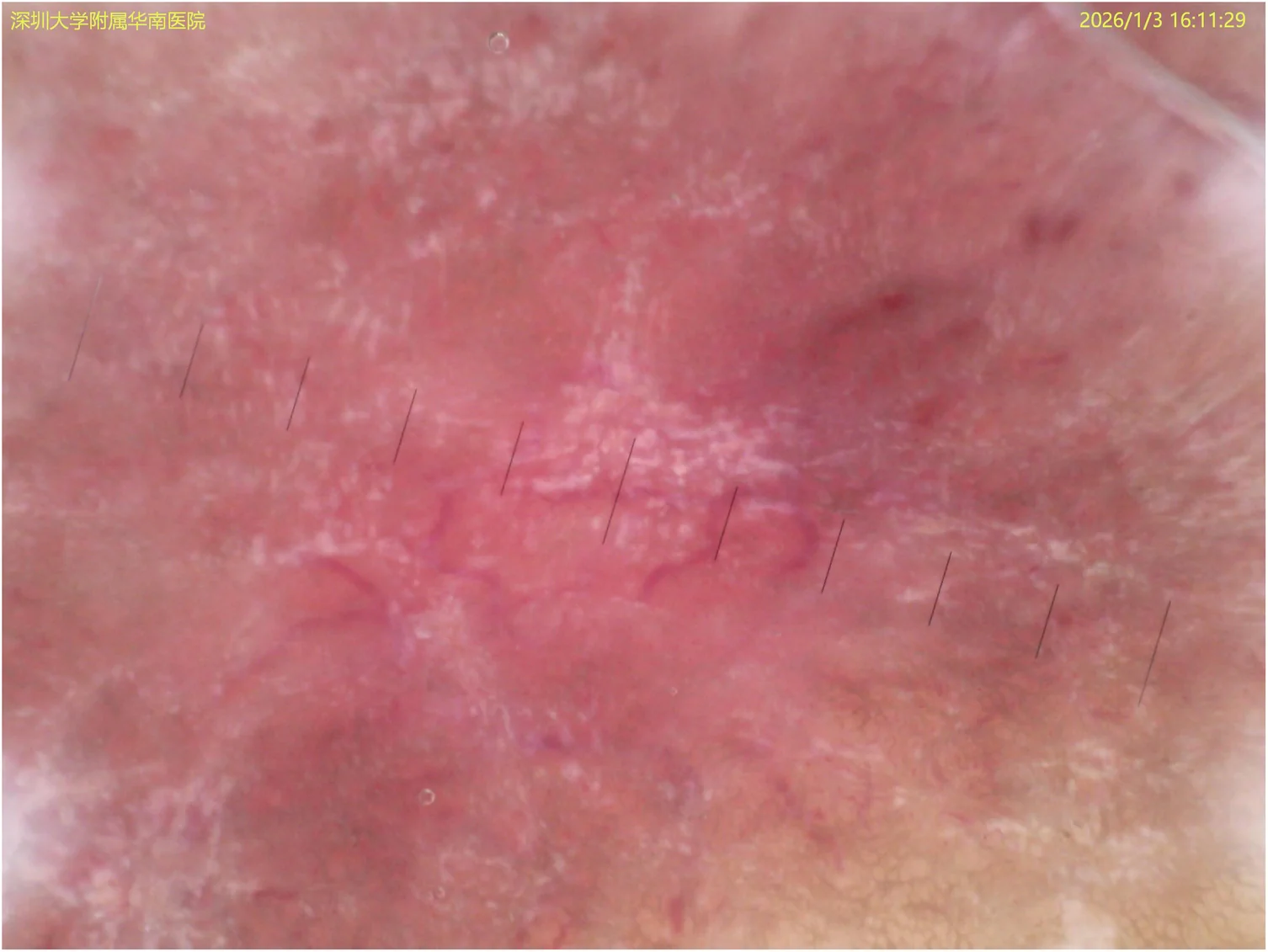
Dermoscopy: pink background, uniform linear vessels, short rod-like shiny white streaks, partially clustered and confluent, without arborizing vessels or no blue-gray ovoid nests.

The original version of this article has been updated.

