# Supplementary figures and images for: Primary cutaneous adenoid cystic carcinoma of the right forearm: a case report and dermoscopic features
Source: Front Med (Lausanne). 2026 Jun 16;13:1872296. doi: 10.3389/fmed.2026.1872296 (PMC13314509; doi:10.3389/fmed.2026.1872296)

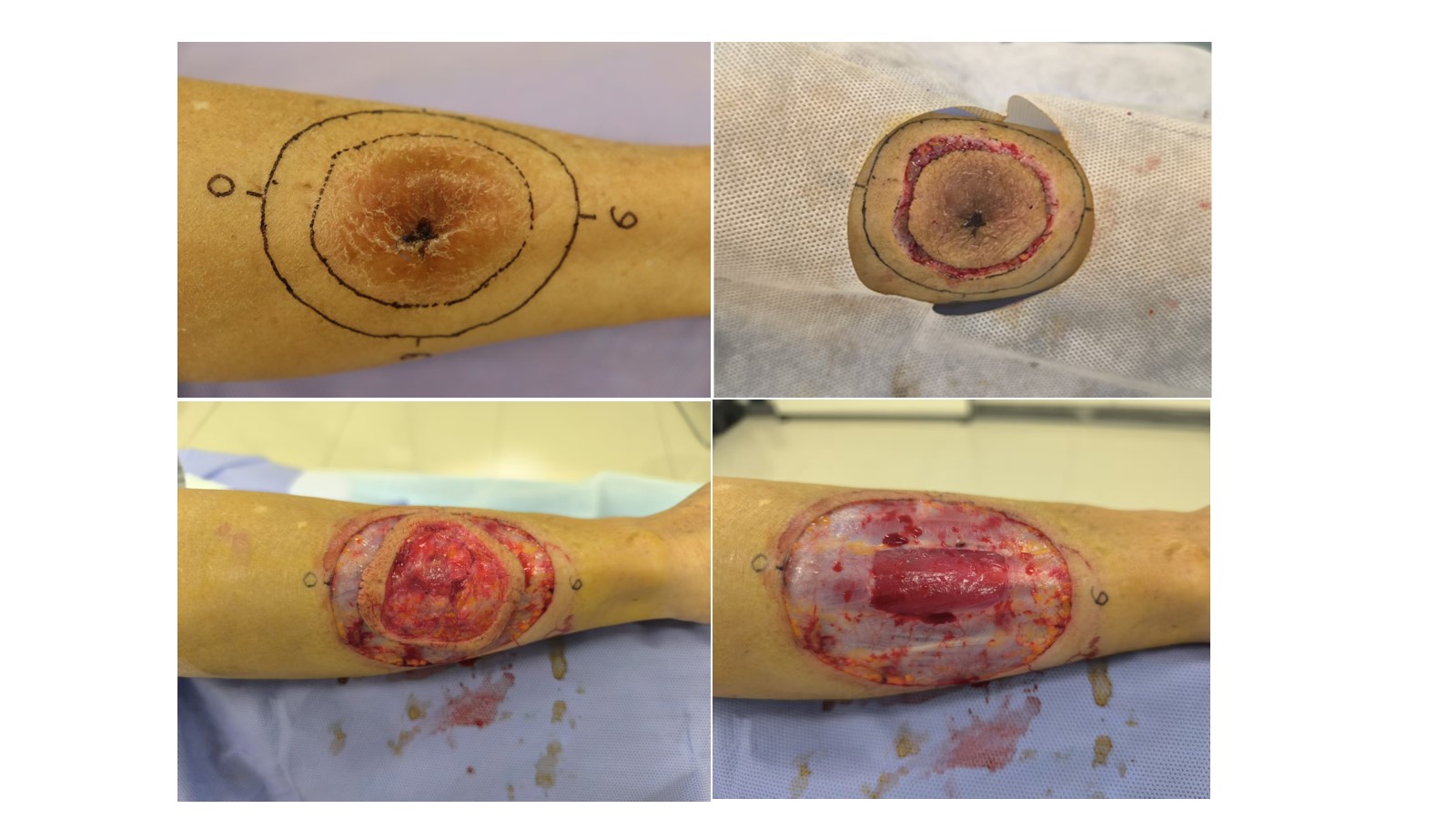

Supplement: Supplementary file 1 [file Image_1.JPEG]
